# Quality and Content of Internet-Based Information for Osteoporosis and Fragility Fracture Diagnoses

**DOI:** 10.5435/JAAOSGlobal-D-20-00192

**Published:** 2021-02-12

**Authors:** Meghan K. Wally, Thomas Bemenderfer, R. Randall McKnight, Jacob D. Gorbaty, Kyle Jeray, Rachel B. Seymour, Madhav A. Karunakar

**Affiliations:** From the Department of Orthopaedic Surgery, Atrium Health Musculoskeletal Institute, Charlotte, NC (Dr. Wally, Dr. Bemenderfer, Dr. McKnight, Dr. Gorbaty, Dr. Seymour, and Dr. Karunakar); the Department of Orthopedic Surgery, Prisma Health-Upstate; Greenville, SC (Dr. Jeray); and the University of South Carolina School of Medicine Greenville, Greenville, SC (Dr. Jeray).

## Abstract

**Introduction:**

We aimed to assess the quality and content of websites addressing orthopaedic conditions affecting older adults, emphasizing osteoporosis and fragility fracture.

**Methods:**

Ten diagnoses were chosen. The transparency of information was assessed via the Health On the Net (HON) score; information content was assessed via diagnosis-specific grading templates. A total of 140 websites (14 per diagnosis) were reviewed by three raters. HON scores and information quality were compared by diagnosis, website type, and website source. The correlation between HON score and information quality score was calculated.

**Results:**

Most websites were commercial (59.3%). Cronbach alpha for Hall scores exceeded the a priori threshold of 0.7. Analysis proceeded using averages across raters. HON score was significantly associated with higher content scores (r = 0.56; *P* < 0.0001). Content scores ranged from 21.1 to 59.4. Content scores differed significantly by diagnosis (*P* = 0.0008) and website source (*P* < 0.0001).

**Discussion:**

The quality and content of websites is highly variable for osteoporosis and fragility fracture diagnoses. Patients should be encouraged to access reputable sites, including sites displaying a HON seal. Academic and medical specialty societies demonstrate opportunity for improvement of their own websites and might be able to lead efforts to increase accessibility of high-quality content.

It is estimated that approximately 10% of the US population aged 50 years and older have osteoporosis and 43.9% have osteopenia, placing them at risk of osteoporosis.^1^ A major concern among older adults, especially those with osteoporosis, is fragility fracture. Fragility fracture typically includes vertebral compression fracture, proximal femur fracture, distal radius, and proximal humerus fractures.^2^ Fragility fracture can lead to decline in functional status, loss of independence, chronic pain, poor psychological and cognitive health, and mortality.^3^ These conditions will burden the US population and healthcare system as the population ages. By 2050, it is estimated that the annual incidence of hip fractures will be 6.3 million.^3^ The Internet is now a common tool for accessing health and medical information, with approximately 60% of US adults accessing this type of information.^4^ Among older adults specifically, 52% of adults aged 65 years or older used the internet, and 30% used the Internet to access health information in 2012.^4^ That percentage has likely risen since because this demographic is the fastest growing group of Internet users.^5^

The content of websites on orthopaedic conditions, including trauma and osteoporosis, is inadequate. A systematic review of studies from 2010 through 2015 assessing information content and readability of websites for orthopaedic conditions identified 38 studies and concluded that information quality and readability is poor.^6^ Literature identified since 2015 consistently found content to be of low-to-moderate quality.^7–9^ No studies have assessed information content on fragility fractures specifically; however, some recent studies have investigated orthopaedic trauma, including clavicle fractures, scaphoid fractures, distal radius fractures, and pelvic/acetabulum fractures and found information to be poor.^10,^11^,^12^,13^ The literature on content of osteoporosis websites was mostly published before 2010. These findings are likely outdated, given the rapid change in Internet content but consistently found information to be of inadequate quality.^14–16^ Joshi et al.^17^ assessed osteoporosis websites in 2011 and concluded that content was of poor quality. Fuzzell et al.^18^ recently assessed internet content related to diphosphonate treatment for osteoporosis and found that only a third of websites had sufficient or accurate information. However, we think that a review of content related to osteoporosis in general, rather than limited to diphosphonate treatment, is warranted.

Knowing how to identify high-quality information on the internet could help patients be good consumers of online content. Some studies have found that academic websites have highest scores, whereas others find government or nonprofit websites to score better.^10,11^ Commercial sites consistently score worse than these.^6,14,17^ Finally, some evidence indicates the higher Google places the website in the search results, the better the quality, indicating that their algorithm may favor high-quality sites.^14^ However, this means that the correct search terms must be used to access the best information.^12^

The quality of current Internet-based information for orthopaedic conditions affecting primarily older adults, such as osteoporosis and fragility fracture, is unknown. The purpose of this study was to assess the quality and content of websites for these diagnoses and compare the quality by website type. We hypothesized that information on the websites would be incomplete and of low quality but that there would be variation by type of website.

## Methods

### Data Collection

A list of orthopaedic fragility fractures with the greatest incidence was compiled for inclusion in the study. The list was finalized based on recommendation by two board-certified orthopaedic surgeons (M.A.K./K.J.). The selected diagnoses were osteoporosis in men, osteoporosis in women, age-related pathologic fracture, hip fracture, femoral neck fracture, intertrochanteric fracture, distal radius fracture, thoracic vertebral compression fracture, lumbar vertebral compression fracture, and proximal humerus fracture.

The methods were modeled after a similar study that assessed the quality and content of website information for orthopaedic sports medicine diagnoses.^19^ After selecting the diagnoses for inclusion, a grading template was created for each diagnosis. The grading template included the type of website, quality, and information content. Website type was categorized as commercial, academic, physician/group, or nonprofit. Websites affiliated with a university, a medical journal, or a medical society were considered academic. Websites that were funded by industry, included advertisements, or sold products were considered commercial. Professional websites for either individual physicians or physician groups (unaffiliated with an academic institution) were classified as physician/group. Finally, websites for organizations funded by government funding or donations were considered nonprofit. If a website did not fit into one of these categories, it was listed as unidentified.

Four common clinical reference websites were also selected and reviewed for all diagnoses (Medscape, American Academy of Orthopaedic Surgeons [AAOS], Physician: Up-to-Date, and Patient: Up-to-Date). These four websites were selected because they represent peer-reviewed clinical references for (1) patients, (2) general providers, and (3) orthopaedic providers. To identify additional websites, Google was selected as the search engine because it is the most commonly used search engine among orthopaedic patients^20^; thus, it should simulate results our patient population would see. The first 10 consecutive, nonduplicative, nonpreselected websites were included in the study and independently reviewed by three authors. This was based on data that internet users typically only check results on the first page and think that the top-ranked result is best.^21,22^ This produced a total of 140 unique websites for review (14 websites for each diagnosis).

### Quality and Health On the Net Code

Quality was evaluated using the Health On the Net (HON) Foundation criteria.^23^ These criteria were developed in 1996 with the goal of improving the quality of information found online. HON measures transparency of information. If desired, website owners may wish to display the HON code seal to document compliance with the HON criteria, and these websites are subject to audits to ensure compliance. We used a 16-point scale previously developed by the senior author to objectively assess compliance with the following HON code principles ^19^: transparency and honesty, authority, privacy and data protection, updating of information, accountability, and accessibility. The full grading criteria may be found in the previous study.^19^

### Information Content

Custom grading templates were developed for each specific diagnosis to assess the accuracy and completeness of information content. These grading templates were modeled after Soot et al.^24^ and Beredjiklian et al.^25^ to include the following domains: disease summary, pathogenesis, diagnostics, treatments, complications, and prevention/prognosis. The maximum possible score for this section was 100 points, weighted accordingly: 20 points for disease summary, 15 points for pathogenesis, 15 points for diagnostics, 20 points for treatment, 15 points for complications, and 15 points for prevention/prognosis.

### Statistical Analysis

After all raters assessed each website, raw data were observed to assess inter-rater reliability. Cronbach alpha was calculated for the three raters together for HON score, information content score, and each information content domain. An a priori threshold of 0.7 was set to support sufficient inter-rater reliability. After documentation of sufficient inter-rater reliability, analysis was conducted with the average of the three raters for both HON and information content scores. For ease of interpretation given, both HON and information content scores were converted to the percentage of the maximum possible score; thus, they are both on a 0 to 100 scale. The mean score and standard deviation for both HON scores and information content were calculated. HON and information content scores were compared by diagnosis, website type, and website source using analysis of variance, whereas the percentage of high-quality websites was compared using chi-square tests. The correlation between HON score and information content score was also calculated using Pearson correlation coefficient. Significance was set at 0.05, and all analyses were conducted using SAS software, Version 9.4 (SAS Institute).

## Results

### Interrater Reliability

**Figure 1:**
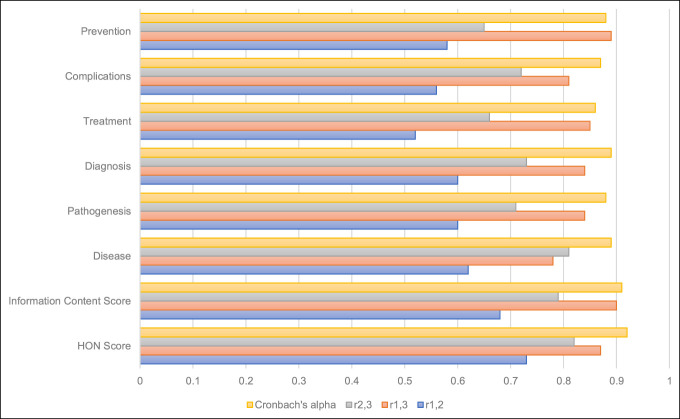
Inter-rater reliability of HON score and information content scores of websites addressing common orthopaedic conditions/injuries. Acceptable threshold for Cronbach alpha set at 0.7. All correlations statistically notable at alpha = 0.05. HON = Health On the Net

Figure 1 displays inter-rater reliability statistics for HON and Information Content scores. Cronbach alpha was calculated for the three raters together, and all exceeded the a priori threshold of 0.7. Pearson correlation coefficients are displayed for each combination of three reviewers. All correlations were statistically \ with an alpha of 0.05. Therefore, analysis proceeded using the average scores across the three raters.

### Descriptive Analyses

Among the 140 websites assessed, most were commercial (59.3%), followed by physician/group (20.0%), nonprofit (10.7%), academic (8.6%), and unidentified (1.4%). Table 1 shows the mean information content and HON scores by diagnosis, website source, and website type.

**Table 1:** Comparison of Information Content Score by Diagnosis, Website Source, and Website Type

Website Characteristic	N	Information Content Score	
		Mean (SD)	*P* Value
Diagnosis			0.0008^a^
Osteoporosis in men	14	47.8 (15.7)	
Osteoporosis in women	14	48.2 (16.7)	
Age-related pathologic fracture	14	21.1 (28.3)	
Hip fracture	14	46.9 (16.2)	
Femoral neck fracture	14	42.7 (22.3)	
Intertrochanteric fracture	14	49.8 (23.8)	
Distal radius fracture	14	40.6 (22.6)	
Thoracic vertebral compression fracture	14	51.7 (20.4)	
Lumbar vertebral compression fracture	14	59.4 (16.1)	
Proximal humerus fracture	14	44.4 (14.3)	
Website source			<0.0001^a^
Google	100	39.0 (19.5)	
Medscape	10	69.8 (11.7)	
American Academy of Orthopaedic Surgeons	10	50.3 (12.9)	
Physician: Up-to-Date	10	79.9 (13.1)	
Patient: Up-to-Date	10	43.6 (10.3)	
Website type			0.8545
Commercial	83	44.5 (24.9)	
Academic	12	47.6 (16.3)	
Physician/group	28	44.9 (13.4)	
Nonprofit	15	45.7 (19.5)	
Unidentified	2	61.5 (19.1)	

a Statistical significance with alpha = 0.05.

Information content scores are presented as the average percentage (and SD) of the maximum possible score.

### Information Content Scores

A statistically significant difference was noted in information content scores by diagnosis (*P* = 0.0008) and website source (*P* < 0.0001). Information content scores ranged from 21.1 (age-related pathologic fractures) to 59.4 (lumbar vertebral compression fracture). According to website source, Physician-facing Up-to-Date sites (79.9) had the highest scores, whereas Google had the lowest (39.0). No significant difference exists by website type (ie, commercial, academic) (*P* = 0.85).

**Figure 2:**
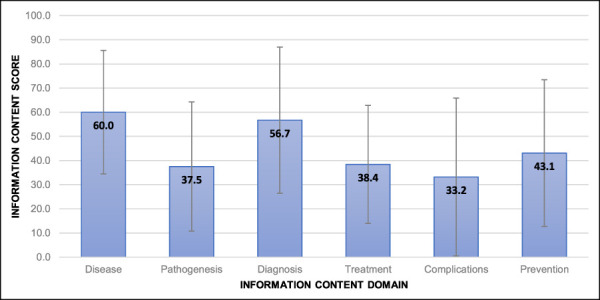
Mean information content score by the information content domain. The values are presented as the mean percentage (and SD) of the maximum possible score for each domain.

Figure 2 displays the mean information content score by domain, for all diagnoses. Information regarding complications had the lowest quality score (33.2), whereas generic information about the disease had the highest quality score (60.0). No statistically notable difference was found in the scores by domain, however.

### Quality and HON Code

A statistically significant difference existed in HON scores by diagnosis (*P* = 0.004), website source (*P* < 0.0001), and website type (*P* < 0.001). HON scores ranged from 42.3 (age-related pathologic fractures) to 72.8 (lumbar vertebral compression fracture). According to website source, Physician-facing Up-to-Date sites had the highest score (87.9), whereas Google had the lowest (58.4). By website type, commercial sites had the highest scores (20.6), whereas academic websites had the lowest (9.6). Table 2 compares the HOM score by diagnosis, website source, and website type.

**Table 2:** Comparison of HON Score by Diagnosis, Website Source, and Website Type

Website Characteristic	N	HON Score		High-Quality Sites According to HON Score	
		Mean (SD)	*P* Value	N (%)	*P* Value
Diagnosis			0.004^a^		0.9
Osteoporosis in men	14	66.8 (15.8)		5 (35.7)	
Osteoporosis in women	14	66.4 (13.5)		5 (35.7)	
Age-related pathologic fracture	14	42.3 (26.4)		3 (21.4)	
Hip fracture	14	69.5 (15.0)		7 (50.0)	
Femoral neck fracture	14	67.3 (17.9)		5 (35.7)	
Intertrochanteric fracture	14	62.5 (19.2)		4 (28.6)	
Distal radius fracture	14	61.5 (19.7)		5 (35.7)	
Thoracic vertebral compression fracture	14	61.1 (22.5)		5 (35.7)	
Lumbar vertebral compression fracture	14	72.8 (13.5)		7 (50.0)	
Proximal humerus fracture	14	67.7 (17.3)		5 (35.7)	
Website source			<0.0001^a^		<0.0001
Google	100	58.4 (19.9)		25 (25.0)	
Medscape	10	81.0 (2.3)		10 (100.0)	
American Academy of Orthopaedic Surgeons	10	63.1 (4.7)		0 (0.0)	
Physician: Up-to-Date	10	87.9 (2.4)		10 (100.0)	
Patient: Up-to-Date	10	77.1 (3.7)		6 (60.0)	
Website type			<0.001^a^		<0.0001
Commercial	83	69.5 (20.6)		47 (56.6)	
Academic	12	41.3 (9.6)		0 (0)	
Physician/Group	28	60.2 (10.8)		0 (0)	
Nonprofit	15	56.1 (17.2)		3 (20.0)	
Unidentified	2	69.8 (10.3)		1 (50.0)	

HON = Health On the Net

a Statistical significance with alpha = 0.05. A high-quality score was one with a HON score of >12 points, which is >75% of the maximum possible score of 16.

HON scores are presented as the average percentage (and SD) of the maximum possible score.

We also compared the percent of websites deemed to be of high quality, as defined by a HON score of ≥12 points, which is ≥75% of the maximum possible score. No difference was observed in percent high quality by diagnosis (*P* = 0.9), but it ranged from 21.4% of websites on age-related pathologic fracture being high quality to 50.0% of websites on hip fracture and lumbar vertebral compression fractures. A significant difference was noted in high-quality websites by website type and source (*P* < 0.001). According to the website source, MedScape and Physician: Up-to-Date were 100% high quality, as compared with 60% for Patient: Up-to-Date, 25% for Google, and none of the AAOS websites. By website type, more than half of commercial websites were of high quality, as compared with 20% of nonprofit websites and none of the academic or physician websites.

**Figure 3:**
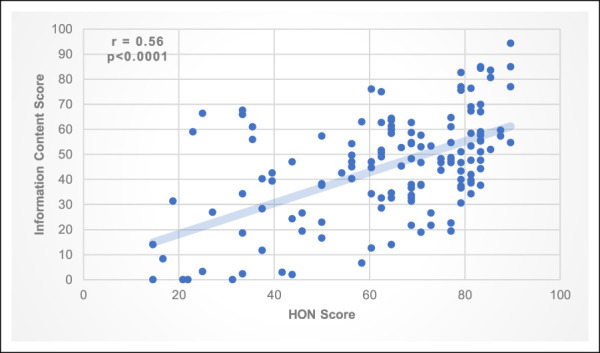
Correlation between HON score and information content score of websites addressing common orthopaedic conditions/injuries. HON = Health On the Net

Figure 3 depicts the correlation between HON Score and Information Content scores. A moderate positive correlation exists between HON Score and Information Content score that is statistically significant (r = 0.56; *P* < 0.0001).

## Discussion

This study is the first attempt to systematically evaluate common websites addressing osteoporosis and fragility fracture diagnoses affecting older adults for quality and accuracy. We found that these websites generally had incomplete or inaccurate information content (between 21% and 59% of maximum score). Only two conditions (thoracic vertebral compression fracture and lumbar vertebral compression fracture) averaged scores more than 50%. Between 21% and 50% of websites were deemed high quality based on HON scores. It is important to note that the HON score does not assess the information content but rather broad principles for ethical provision of information (ie, transparency and accountability).

Many studies use the JAMA benchmark criteria,^26^ the DISCERN criteria,^27^ or the Health On the Net Code (HON Code)^23^ to standardize grading of website quality. In a systematic review of websites on orthopaedic conditions, between 4% and 44% of websites were deemed “high quality” based on one of these standardized grading tools.^6^ Recent studies assessing content on orthopaedic trauma found information to be poor (between 29% and 47% of maximum quality scores).^10,^11^,^12^,13^ This study found HON scores ranging from 42% to 73% of maximum scores, and between 21% and 50% of websites were deemed high-quality based on HON score. This suggests that websites on fragility fracture and osteoporosis are of similar or slightly better quality compared with other orthopaedic websites. Regarding condition-specific information content (ie, completeness and accuracy), a systematic review found scores ranged between 38% and 70% of the total scores.^6^ This study found information content scores to range between 21% and 59%, suggesting that the content for fragility fracture and osteoporosis is slightly worse than other orthopaedic websites.

Our results confirm the findings of previous studies that commercial websites are the most common, representing 60% in this study. Although concern exists for bias related to industry funding or direct sales, the HON scores for these sites were generally high, indicating transparency. However, patients may not be knowledgeable enough to discern potential biases. Despite the proliferation of health information on the Internet and prevalence of patients accessing the Internet for this information, we did not see a large increase in the number of sites deemed high quality according to HON score (36%) compared with previous studies.^6,19^ We did observe a modest yet statistically notable correlation between HON Score and information content; thus, HON score may be useful for identifying websites with complete and accurate information. This association is consistent with most previous studies.^6,11,19,28,^29^,^30^,31^ Furthermore, Physician: Up-to-Date and Medscape were consistently reliable resources based on the HON criteria. Surgeons may consider directing patients to these websites or looking for sites displaying the HON code seal of compliance. It is unknown to what extent patients are aware of the HON criteria or use them to judge websites. One study found that patients primarily judge the credibility of a website based on the source, a professional design, language, and ease of use yet rarely read about the authors or check for disclaimers or disclosure statements.^22^

Although Cassidy and Baker^6^ found better information on academic, subspecialty society, and nonprofit websites, our results do not support one type of website over another based on the information content scores. Comparing results between our study and these is challenging because of the variety in measures used. Many used a content score specific to the diagnosis of interest, as did we, to assess information content. In addition, their systematic review did not identify or include any studies assessing the same conditions or diagnoses as our study. It could be that academic and subspecialty societies do a poor job of addressing fragility fracture and osteoporosis. Regarding quality, our results indicate HON scores to be highest for commercial groups, whereas Cassidy and Baker^6^ found higher quality websites (as measured by HON, DISCERN, and JAMA benchmark criteria) for academic, subspecialty, and nonprofit websites. It is possible that the criteria judged by the DISCERN and JAMA benchmark criteria are more likely to be present on academic or subspecialty sites. Specifically, JAMA benchmark assesses authorship, attributions, disclosure, and currency. Academic and subspecialty sites may be more likely than commercial sites to have these components, despite the accuracy or completeness of information content. Only four of the studies assessing relationship between website authorship and reliability included HON Code to compare directly with our results.^19,29,32,33^ Of these, only Starman et al.^19^ reported HON Code by website authorship, and they found nonprofit websites to score highest. They found 25% of academic sites to be of high quality based on the HON Code and only 4.5% of physician websites. Our study found no academic or physician websites that were of high quality. In particular, the AAOS did poorly in our study. Previous research has acknowledged that AAOS content is also at a high reading level that makes it inaccessible to most patients.^34^ Based on our results, clinicians have the opportunity to effect change by improving the content of academic and specialty societies websites.

We found websites identified by a search engine to have *both* the lowest HON scores and information content scores. However, most orthopaedic patients use a search engine to find information. One study found that 14% of patients followed their surgeon's advice for locating online content, rather than a search engine. When patients accessed sites recommended by their surgeon, they reported statistically notably higher quality of information compared with search engines.^35^ Patients are generally unsatisfied with information found online because of unreliable information, inability to find information, or inability to understand the content.^36^ Most orthopaedic patients were skeptical of information found online (68%) and would welcome surgeon's guidance on which internet sources to use (83%).^37^ Furthermore, most continue to prefer verbal communication with their surgeon to learn about their condition.^36^ As a supplement to communication with their surgeon, however, more preferred a website as opposed to a paper handout.^36^ This highlights notable opportunity for surgeons to participate in patient education regarding consumption of online orthopaedic content. Surgeons should be aware of information available on the internet because their patients will likely access it.

Our study is unique because we also assessed individual content domains. In general, the disease and diagnosis were most consistently well covered; however, the average scores were only 60% and 57%, respectively, indicating notable room for improvement. Notably, complications were least well covered (33%), although this may be a notable concern for patients after returning home, when they are likely to turn to the Internet for information.

Despite the fact that information on the Internet is generally poor, patients and their caregivers will continue to access and use this information. When information is incomplete or misleading, patients may have unrealistic expectations regarding recovery and thus poorer satisfaction. On the other extreme, inaccurate information may cause a patient to disregard their surgeon's treatment instructions (such as weight-bearing restrictions) or delay seeking care for a notable complication. It is important, therefore, to counsel patients regarding where to source information on the internet proactively.

Several limitations of this study exist. The grading of information content is subjective; however, we attempted to standardize the grading criteria, and our interrater reliability analysis demonstrates acceptable consistency in grading. In addition, the grading sheets differed for each diagnosis, which may limit our ability to compare scores between diagnoses. However, we did apply a standard grading criteria for quality, the HON Code, and assessed interobserver reliability, which were limitations of many previous studies.^6^ Although we used the most common search engine, we do not know whether our search methodology is representative of the typical methods used by our patient population, particularly among older adults (ie, use of a different search engine, different search terms, or choosing websites not by the first to appear, but some other strategy). Finally, we did not assess readability, which may be a barrier to the utility of these websites, even if the content and quality are excellent.^6^ In fact, many studies have found an association between higher quality and lower readability, particularly society websites, including AAOS.^6,34^ Engaging experts in health literacy in content development or supplementing written material with videos may improve accessibility. However, this study was the first to systematically assess the content of websites for these conditions and highlights areas of opportunity for improvement in Internet-based information and practical suggestions for counseling patients proactively on their use of health-related websites. Although we identified statistically notable differences in information content and quality, it is unknown what difference in the information content scores or HON scores represents a minimum clinically important difference.

In summary, the quality and content of websites is highly variable for common osteoporosis and fragility fracture topics. Patients should be encouraged to access reputable sites, and orthopaedic surgeons may consider suggesting sites displaying the HON seal to improve patient knowledge and, ultimately, promote shared decision-making. We encourage academic and medical specialty societies to show leadership in this arena by improving the content of their own patient-facing materials and websites.
